# Characteristics of pediatric COVID-19 infections and the impact of influenza and COVID-19 vaccinations during the first two years of the pandemic

**DOI:** 10.3389/fped.2023.1046680

**Published:** 2023-10-12

**Authors:** Mahmoud Ali, Lynette Phillips, David C. Kaelber, Hulya Bukulmez

**Affiliations:** ^1^ The Department of Pediatrics, The MetroHealth System, Case Western Reserve University, Cleveland, OH, United States; ^2^ College of Public Health, Kent State University, Kent, OH, United States; ^3^ The Departments of Internal Medicine and Population and Quantitative Health Sciences and the Center for Clinical Informatics Research and Educations, The MetroHealth System, Case Western Reserve University, Cleveland, OH, United States

**Keywords:** coronavirus, COVID-19, multisystem inflammatory syndrome in children (MIS-C), body mass index (BMI), obesity, influenza, vaccine

## Abstract

The data regarding the demographics of SARS-CoV-2 in the pediatric population has been published based on several single-center experiences or on metanalyses over short time frames. This article reports data on the demographics of pediatric patients with COVID-19 on a global scale using the TriNetX COVID-19 Research Network. In addition, we examined the risk of COVID-19 infection in relation to the body mass index (BMI) category and the protective value of influenza and COVID-19 immunization against COVID-19 infection. The incidence of COVID-19 infection was higher in the younger age group (≤6 years old), but no gender differences. The incidence of COVID-19 infection was higher among African Americans/Black race (28.57%) White race (27.10%), and obese patients; across all age groups, all genders, all races, and ethnicities (*p* < 0.0001). The incidence of MIS-C was also higher in patients with obesity (OR 1.71, CI 1.36–2.14). We found that the patients who were neither vaccinated for COVID-19 nor influenza within one year before their COVID-19 diagnoses compared to those who received influenza vaccine only, had significantly higher odds for hospitalization (OR 1.19, CI 1.18–1.21), development of MIS-C (OR 1.52, CI 1.32–1.74), and more importantly mortality (OR 1.47, CI 1.26–1.71). In addition, those patients who were neither vaccinated for COVID-19 nor influenza within one year before their COVID-19 diagnoses, compared to those who received at least one dose of COVID-19 vaccine, had significantly higher odds for hospitalization (OR 1.11, CI 1.04–1.19). However, those patients who did not receive the influenza vaccine within one year before their COVID-19 diagnoses nor received the COVID-19 vaccine had much higher odds for hospitalization (OR 1.46, CI 1.41–1.51), MIS-C (OR 3.72, CI 2.11–6.56), and mortality compared to those who received both vaccinations (OR 13.55, CI 1.91–9.62). Using the multiplicative interaction scale, we found a positive interaction between the COVID-19 vaccine and the influenza vaccine; they both combined have a larger effect than each separately. Our study is the largest of its kind (to date) examining the global demographic of the pandemic and the first of a kind to find a link between influenza vaccine and COVID-19-related hospitalization, MIS-C, and mortality in the pediatric population.

## Introduction

Severe acute respiratory syndrome coronavirus 2 (SARS-CoV-2) spanned the earth in less than four months from its inauguration in Wuhan, China, in December 2019, resulting in the World Health Organization (WHO) announcing it as a pandemic on March 11, 2020 (1). To date, approximately 565 million individuals (resulting in more than six million deaths, in 215 countries, areas, or territories have been affected (2). As of July 22, 2022, more than eighty-nine million cases of COVID-19 have been confirmed in the USA (3). A recent report from the American Academy of Pediatrics and the Children's Hospital Association estimated nearly 13.5 million COVID-19 cases among children, with 0.1%–1.5% of these children being hospitalized, and a very low mortality rate (<0.02%) (3). Previous studies have shown that age, gender, and race/ethnicity are independent risk factors for poor outcomes in COVID-19 (4–7). Obesity was reported as an independent risk factor for critical illness in pediatric patients with COVID-19, and this association was modified by age (8). Also, obese communities were found among the best predictors of COVID-19-related deaths (9). The goal of our work is to describe—at a significantly larger scale—the distribution of COVID-19 among different pediatric age groups, races, ethnicities, BMI categories, and the relation between multisystem inflammatory syndrome in children (MIS-C) incidence (being one of the severe COVID-19 complications) and BMI, as well as the relationship between prior influenza and/or COVID-19 vaccination, and hospitalization, MIS-C, and mortality. Understanding how these factors play a role in infection with SARS-CoV-2 is crucial for patient risk stratification and care, and will potentially enlighten researchers and clinicians about which children are at higher risk for developing COVID-19 infection and its secondary complications.

## Methods

In this retrospective cohort analysis, data analysis was performed using the TriNetX COVID-19 Research Network. TriNetX is a real-world platform of aggregated electronic health records (EHRs) from seventy-five health care organizations (HCOs) globally, which has a total of 17,158,646 pediatric patients ages 18 years or less. Two-thirds of the TriNetX patients are from the US and one-third from outside the US including (∼10% Latin America, ∼10% Europe, and ∼10% Asia). We created queries using the TriNetX's analytics function to analyze the outcomes of interest among children up through 18 years old, between December 2019 till July 22, 2022. The outcomes of interest included (1) COVID-19 infection among different age groups, genders, races, ethnicities, and BMI categories, (2) incidence of MIS-C among different age groups and different BMI categories, and (3) odds of hospitalization, MIS-C, and mortality among COVID-19 patients according to their COVID-19/influenza vaccination status. To examine the association between exposure and outcome, we used the TriNetX's analytics function to perform propensity score matching and calculate the odds ratio (OR) for each outcome of interest. Regression analysess were not performed.

The data accessible through TriNetX as been attested to being de-identified through a formal determination by a qualified expert as defined in Section 164.514(b)(1) of the HIPAA Privacy Rule. Because of this determination, we did not seek, nor did we obtain, Institutional Board Approval for this research. TriNetX also offers built-in analytics, charts, graphs, and statistical options for data visualization. We stratified the patient cohort into three different age groups: Up to 6 years old, between 7 and 12 years, and between 13 and 18 years old. We also stratified the population based on weight by ICD codes normal weight (ICD 10: Z68.52), overweight (ICD 10: Z68.53), and obese (ICD 10: Z68.54). Furthermore, we created queries of patients who received influenza vaccination within one year of the outcome of interest (hospitalization, MIS-C diagnosis, or death), or COVID-19 vaccination, or both influenza vaccination and at least one COVID-19 vaccination before these outcomes. Visit codes were used for hospitalization/admission, ICD encounter diagnosis codes were used to identify MIS-C, and patient status codes were used to mortality.

Race categories included American Indian or Alaskan Native, Asian, African American/Black, Native Hawaiian or other Pacific Islander, White, and unknown. Ethnicity categories included Hispanic or Latino, Not Hispanic/Not Latino, and unknown. We used the TriNetX codes to identify the variables and outcomes of interest (see Supplementary Material). We used built-in analytic functions to calculate the incidence of COVID-19 among different age groups, the odds ratios of COVID-19 and MIS-C among the three weight groups, and the odds ratios of hospitalization, MIS-C diagnoses, and mortality for COVID-19 patients who were vaccinated against influenza, COVID-19, both, or neither. Chi-square tests were used to compare the observed incidence differences between genders, races, and ethnicities across different weight groups to the expected incidences. Multiplicative interaction was assessed for the outcomes of hospitalization, MIS-C, and mortality by dividing the odds ratio for COVID-19 infection for those who received both the influenza vaccine and the COVID-19 vaccine by the product of the odds ratio for the influenza vaccine only multiplied by the odds ratio for COVID-19 vaccine only. A ratio greater than 1.0 indicates positive interaction, whereas a ratio of less than 1.0 signifies negative interaction. An equal ratio of 1.0 represents no interaction on the multiplicative scale.

## Results

### Ethnicity and gender differences in the incidence rate of COVID-19 infection

The incidences of COVID-19 infection in (males/females) in the three age groups we investigated: Up to 6 years, between 7 and 12 years, and between 13 and 18 years old were 26.80/27.63%, 24.21/24.25%, and 23.63/23.09% respectively. The incidence of COVID-19 infection was higher among African American/Black race (28.57%) followed by White race (27.1%), patients with unknown/unreported race 21.22%, Native Hawaiian or other Pacific Islanders 20.92%, American Indian or Alaskan Native 19.92%, and Asian race 19.1%. In addition, the incidence of COVID-19 infection was 27.27% among patients of Hispanic ethnicity vs. 27.37% among non-Hispanic patients; patients with un-reported ethnicity had an incidence of 21.79% (Table 1).

**Table 1:** Incidence of COVID-19 in pediatric population in different gender, race and ethnic background stratified by age group.

Age (years)		Up through 6	7–12	13–18	Total ≤18
Total number of populations (*n*)		4,683,222	5,993,104	6,482,320	17,158,646
COVID-19 patients (*n*) (%)		930,016 (27.16)	673,323 (24.16)	741,477 (23.31)	2,344,816 (24.97)
Gender with COVID-19 diagnosis	Female	427,773 (26.8)	322,458 (24.21)	382,603 (23.63)	1,132,798 (24.91)
	Male	502,010 (27.63)	350,687 (24.25)	358,565 (23.09)	1,211,262 (25.15)
	Unknown	269 (2.26)	178 (2.03)	309 (3.57)	756 (2.58)
Race with COVID-19 diagnosis	American Indian or Alaskan native	4,033 (21.83)	2,859 (19.17)	3,179 (18.53)	10,107 (19.92)
	Asian (*n*) (%)	21,666 (22.73)	14,507 (18.45)	12,482 (15.45)	48,655 (19.1)
	African American/Black	158,110 (33.05)	102,385 (26.84)	105,964 (25.06)	366,459 (28.57)
	Native Hawaiian or other Pacific Islander	953 (20.29)	805 (22.63)	874 (20.21)	2,632 (20.92)
	Unknown	293,086 (22.86)	199,045 (20.26)	210,061 (20.11)	702,192 (21.22)
	White	452,168 (29.25)	353,686 (26.68)	408,917 (25.39)	1,214,771 (27.1)
Ethnicity with COVID-19 diagnoses	Hispanic or Latino	150,727 (31.15)	96,252 (25.97)	95,775 (23.81)	342,754 (27.27)
	Not Hispanic/not Latino	443,992 (30.55)	325,441 (26.43)	354,782 (24.94)	1,124,215 (27.37)
	Unknown ethnicity	335,297 (22.54)	251,630 (21.24)	290,920 (21.46)	877,847 (21.79)

≤, less than or equal to; %, percent; *n*, number.

### Obesity increases the risk of developing COVID-19 infection in children of Native Hawaiian race and Hispanic or Latino ethnic background

The incidence of COVID-19 infection among normal weight, overweight, and obese patients were statistically different; 30.32%, 31.33%, and 31.91%, respectively, *p* < 0.00001. The distribution of all the ICD encounter diagnoses BMI categories in the general population (5th to <85th, 85th to <95th, and 95th or above) were similar to the distribution of the three BMI categories in the patients diagnosed with COVID-19 diagnoses among the same BMI categories (48.00(?)%, 18.00(?)%, 32.50(?)% vs. 47.59%, 18.89%, 33.52%). When analyzed using race, obesity increased the incidence of COVID-19 only among Native Hawaiian patients (45.13%). However, African American (38.26%), American Indian or Alaskan Natives (34.8%), and White (30.62%) patients, obesity did not increase the risk of COVID-19 infection. When analyzed by ethnic backgrounds, obesity diagnosis increased the risk of COVID-19 diagnosis among Hispanics/Latino population, 42.99% of but did not among Non-Hispanic/Non-Latino population 28.63% (*p* < 0.00001). A majority of patients that were Non-Hispanic or Non-Latino who developed COVID-19 infections had normal weight diagnoses (52.51%) as compared to the Hispanic and Latino ethnic backgrounds (36.24%). Most patients who have COVID-19 diagnosis and with unknown ethnicity (unreported), did not have an obesity diagnosis—45.16% had normal BMI, 19.91% were overweight, and 34.94% were obese. The results reflect that obesity as a suggested risk factor for COVID-19 infection should be interpreted in the context of racial and ethnic backgrounds (Table 2).

**Table 2:** Incidence of COVID-19 in pediatric population stratified by weight category.

Weight category		Normal weight *n* (percent)	Overweight *n* (percent)	Obese *n* (percent)
Number in the general population with weight category reported (*n* = 1,203,905)		586,341 (48%)	225,213 (18%)	392,351 (32.50%)
Incidence rate of COVID-19 diagnosis among patients (*n* = 373,536)		30.32%	31.33%	31.91%
Distribution of weight categories in patients with COVID-19 diagnosis (*n* = 373,536)		177,778 (47.59%)	70,559 (18.89%)	125,199 (33.52%)
Gender	Female (*n* = 162,745)	78,206 (48.05%)	32,460 (19.95%)	52,079 (32%)
	Male (*n* = 169,134)	81,400 (48.13%)	31,078 (18.38%)	56,656 (33.50%)
	Unknown (*n* = 45)	21 (46.67%)	10 (22.22%)	14 (31.11%)
Race	American Indian or Alaskan native (*n* = 1,618)	718 (44.37%)	337 (20.83%)	563 (34.8%)
	Asian (*n* = 8,874)	5,310 (59.84%)	1,623 (18.29%)	1,941 (21.87%)
	African American/black (*n* = 64,985)	26,830 (41.29%)	13,289 (20.45%)	24,866 (38.26%)
	Native Hawaiian or other pacific islander (*n* = 421)	149 (35.39%)	82 (19.48%)	190 (45.13%)
	Unknown (*n* = 67,774)	31,306 (46.19%)	12,928 (19.07%)	23,540 (34.37%)
	White (*n* = 188,249)	95,314 (50.63%)	35,286 (18.74%)	57,649 (30.62%)
Ethnicity	Hispanic or Latino (*n* = 60,440)	21,902 (36.24%)	12,553 (20.77%)	25,985 (42.99%)
	Not Hispanic/not Latino (*n* = 205,918)	108,119 (52.51%)	37,941 (18.43%)	59,858 (28.63%)
	Unknown ethnicity (*n* = 65,563)	29,606 (45.16%)	13,051 (19.91%)	22,906 (34.94%)

≤, less than or equal to, %, percent, *n*, number.

### Increasing weight increases the risk of developing COVID-19 infection and MIS-C following COVID-19 infection in children

When adjusting for age, the incidence of COVID-19 was higher in patients with obesity compared to those with normal weight across all age groups (28.67% in patients up to 6 years old, 23.97% in patients between 7 and 12 years old, and 22.29% in patients between 13 and 18 years old) vs. (27.32%, 23.06%, and 21.54%), the odds ratios were 1.07 (95% CI 1.04–1.09), 1.05 (95% CI 1.03–1.07), and 1.04 (95% CI 1.03–1.06) respectively. The incidence of MIS-C was higher in total patients with an obesity diagnosis compared to patients with a normal weight diagnosis (0.041% vs. 0.024%, OR 1.71, CI 1.36,2.14), the higher incidence of MIS-C in obese patients was prominent across all age groups (0.057% in patients up to 6 years old, 0.047% in patients between 7 and 12-years old, and 0.03% in patients between13 and 18 years old) vs. (0.029%, 0.024%, and 0.02) respectively, the odds ratios were 1.95 (95% CI1.19–3.17), 1.98 (95% CI1.37–2.84), and 1.62 (95% CI1.11–2.36) respectively (Table 3).

**Table 3:** Incidence and odd ratios of COVID-19 infection and multisystem inflammatory syndrome in children (MIS-C) stratified by weight category.

Weight category/age (y)		Up through 6	7–12	13–18	Total ≤18
COVID-19 infection	Normal weight (Reference group)				
	*N* of patients	146,232	218,692	221,296	586,220
	*N* of patients with the outcome	39,947	50,434	47,676	138,057
	Incidence %	27.32	23.06	21.54	23.55
	Overweight				
	*N* of patients	37,142	102,415	102,855	242,214
	*N* of patients with the outcome	10,396	23,853	22,839	57,088
	Incidence (%)	27.99	23.29	22.2	23.55
	OR (95% CI)	1.03 (1.00–1.06)	1.01 (0.99–1.03)	1.04 (1.02–1.06)	1.01 (1.00–1.02)
	*P* value	0.38	0.1	<0.0001	0.16
	Obese				
	*N* of patients	45,328	140,160	206,292	391,780
	*N* of patients with the outcome	12,998	33,591	45,992	92,581
	Incidence (%)	28.67	23.97	22.29	23.63
	OR (95% CI)	1.07 (1.04–1.09) ^a^	1.05 (1.03–1.07) ^a^	1.04 (1.03–1.06) ^a^	1.003 (0.99–1.01)
	*P* value	<0.0001	<0.0001	<0.0001	0.105
MIS-C	Normal weight (Reference group)				
	*N* of patients	146,333	218,709	221,272	586,314
	*N* of patients with the outcome	43	52	45	140
	Incidence %	0.029	0.024	0.02	0.024
	Overweight				
	*N* of patients	37,176	102,417	102,849	242,442
	*N* of patients with the outcome	10	36	26	72
	Incidence (%)	0.027	0.035	0.025	0.03
	OR (95% CI)	0.91 (0.46–1.08)	1.48 (0.97–2.26)	1.24 (0.772.01)	1.25 (0.93–1.67)
	*P* value	0.96	0.11	0.25	0.32
	Obese				
	*N* of patients	45,429	140,501	206,273	392,203
	*N* of patients with the outcome	26	66	68	160
	Incidence (%)	0.057	0.047	0.03	0.041
	OR (95% CI)	1.95 (1.19–3.17) ^a^	1.98 (1.37–2.84) ^a^	1.62 (1.11–2.36) ^a^	1.71 (1.36–2.14) ^a^
	*P* value	0.005	0.001	0.002	0.004

≤, less than or equal to; <, less than; %, percent; *n*, number; MIS-C, multisystem inflammatory syndrome in children.

^a^ indicates statistically significant.

### Influenza vaccination, COVID-19 vaccination, either one alone or both significantly improve the outcomes in children with COVID-19 infection

Among COVID-unvaccinated patients, those who did not receive influenza vaccine within one year prior to COVID-19 infection had a higher incidence of hospitalization, MIS-C diagnosis, and death (8.4%, 0.08%, and 0.07%, respectively) compared to those who were influenza-vaccinated (7.14%, 0.06%, and 0.05%), the odds ratio and confidence interval are (OR 1.19, 95% CI 1.1–1.28), (OR 1.52, 95% CI 1.32–1.74), and (OR 1.47, 95% CI 1.26–1.71) respectively (Table 4).

**Table 4:** The odds of hospitalization, MIS-C, and mortality among pediatrics patients according to their COVID-19 /influenza vaccination status.

Unvaccinated against influenza or COVID-19 (*n*)		1,921,341	Incidence (%)	OR CI (95%) *P* value	Unvaccinated against influenza or COVID-19 (*n*)		1,921,341	Incidence (%)	OR CI (95%) *P* value	Unvaccinated against influenza or COVID-19 (*n*)		1,921,341	Incidence (%)	OR CI (95%) *P* value
Influenza vaccinated (*n*)		**422,683**			**Covid-19 vaccinated (*n*)**		**9,824**			**Double vaccinated**		**52,872**		
Unvaccinated	Hospitalization (n)	161,318	8.4	1.19 (1.18–1.21) <0.0001	Unvaccinated	Hospitalization (n)	208,025	10.82	1.11 (1.04–1.19) 0.002	Unvaccinated	Hospitalization (n)	161,318	8.4	1.46 (1.41–1.51) <0.0001
Influenza- vaccinated	Hospitalization (n)	30,192	7.14		Covid vaccinated	Hospitalization (n)	943	9.56		Double vaccinated	Hospitalization (n)	3,127	5.91	
Unvaccinated	MIS-C (n)	1,637	0.08	1.52 (1.32–1.74) <0.0001	Unvaccinated	MIS-	1,691	0.09	0.84 (0.45–1.57) 0.59	Unvaccinated	MIS-C	1,637	0.08	3.72 (2.11–6.56) <0.0001
Influenza vaccinated	MIS-C (n)	235	0.06		Covid vaccinated	MIS-C (n)	10	0.10		Double vaccinated	MIS-C (n)	12	0.02	
Unvaccinated	Mortality (n)	1,290	0.07	1.47 (1.26–1.71) <0.0001	Unvaccinated	Mortality (n)	2,242	0.12	1.12 (0.60–2.08) 0.73	Unvaccinated	Mortality (n)	1,290	0.07	3.55 (1.91–9.62) <0.0001
Influenza vaccinated	Mortality (n)	193	0.05		Covid vaccinated	Mortality (n)	10	0.10		Double vaccinated	Mortality (n)	10	0.02	

Unvaccinated = neither received Influenza vaccine (within 1 year before the outcome), nor received Covid-19 vaccine before COVID-19 diagnoses. Influenza vaccinated = received Influenza vaccine (within 1 year before the outcome) but not Covid-19 vaccine. Covid-19 vaccinated = received Covid-19 vaccine before the outcome but not Influenza vaccine within 1 year before the outcome. Double vaccinated = received both Influenza vaccine (within 1 year before the outcome), and at least one dose of Covid-19 vaccine before the COVID-19 diagnosis. *n*, number; %, percent; <, less than; OR, odds ratio; CI, confidence interval; MIS-C, multisystem inflammatory syndrome in children.

In the same context, among influenza-unvaccinated patients who did not receive COVID-19 vaccine before their COVID-19 diagnosis had higher incidence of hospitalization (10.55%) and development of MIS-C (0.09%) compared to those who received at least one dose of COVID-19 vaccine before their COVID-19 infection (9.56% vs. 0.10%) respectively, the odds ratio and confidence interval are (OR 1.11, 95% CI 1.04–1.19) and (OR 0.84, 95% CI 0.45–1.57) respectively. Similarly, the mortalities were higher (0.11% among COVID-19-unvaccinated compared to 0.10% among COVID-19-vaccinated), the odds ratio and confidence interval are (OR 1.12, 95% CI 0.6–2.08) respectively (Table 4). The incidence of hospitalization (8.4%) and MIS-C diagnosis (0.08%) was higher among patients who were not vaccinated against neither Influenza nor COVID-19 within one year prior to COVID-19 infection compared to patients who were vaccinated against both for Influenza and COVID-19 (5.91% and 0.02% respectively) (OR 1.46, CI 1.41–1.51 and OR 3.72, CI 2.11–6.56 respectively). Similarly, the mortality rate was also found higher in the unvaccinated group (0.07%) as compared to the group vaccinated against both infection (0.02%) (OR 13.55, CI 1.91–9.62). (Table 4). Using the multiplicative interaction calculation, we found a positive interaction between the COVID-19 vaccine and influenza vaccine for all outcomes. While the interaction is modest for hospitalization after COVID-19 infection (ratio = 1.1), the interaction was strong for MIS-C (ratio = 2.9) and mortality (ratio = 2.2) outcomes, indicating that the presence of both vaccinations had a stronger impact than what would be expected by the combination of each vaccine's independent effect.

## Discussion

To understand the true burden of COVID-19, it is critical to consider patient characteristics and their consequences on the public health response. Our study analyzed a global cohort of 17,158,646 pediatric patients, the most extensive data set reported thus far. The key results of our study are: (1) there are no gender differences in the incidence of COVID-19 infection in children, (2) there are no ethnic (Hispanic or Latino/Not Hispanic or Not Latino) differences in the incidence of COVID-19 infection in children, (3) Race (American Indian or Alaskan Native, Asian, African American/Black, Native Hawaiian or other Pacific Islander, White, and unknown) differences are similar to those reported from small cohorts previously with African American/Black patients being at highest risk, (4) COVID-19 infection is more common in younger age groups (up to 6 years old), (5) COVID-19 patients of Native Hawaiian race, and those of Hispanic ethnicity are more likely to be obese, (6) the odds of MIS-C were higher in patients with at least one ICD encounter diagnosis code for overweight or obesity, as compared to patients with a normal weight ICD encounter diagnosis code, (7) patients who were vaccinated against influenza alone, or COVID-19 alone, or vaccinated against both have better outcomes for hospitalization, MIS-C, and mortality compared to patients who are not vaccinated against either Influenza, or COVID-19, or both. Most importantly, we show that having received both COVID-19 and influenza vaccination have a multiplicative protective effect than each separately.

Our study is the largest of its kind (to date) targeting to explore the global demographics of the pandemic using TriNetX network, and the first of its kind analyzing the impact of influenza vaccination alone, COVID-19 vaccination alone, or both vaccination in COVID-19-related disease severity such as hospitalization, MIS-C diagnosis, or mortality among pediatric patients.

Unlike other coronaviruses, SAR-CoV-2 has been recognized to cause a unique post-viral immune reaction, a multisystem inflammatory syndrome in children (MIS-C) (21, 22). MIS-C presents as a critical febrile illness, and patients might be hospitalized in intensive care units and receive respiratory support for weeks. MIS-C has been suggested to significantly increase the risk of death in children, unlike acute infection. One of the theories behind the etiopathogenesis of MIS-C, which was not described in the previous coronavirus outbreaks, is the possibility of a residual viral infection that the host is developing a hypersensitivity reaction against (26). Children are found to shed viral particles in respiratory secretions and stool during acute infection, through the convalescent period and beyond (10–13). Viral particles were found in the respiratory secretions and stool of MIS-C patients (13). Our results indicate that MIS-C by itself is seen less often in vaccinated children. Even a previous vaccination with influenza vaccine seems to help protect children from developing MIS-C, an observation which is supported by evolving literature; Domnich et al., in an observational study found a protective effect of influenza vaccine against COVID-19 infection (16). Similarly, a recent meta-analysis reported lower risk of SARS-CoV-2 infection among influenza vaccinated participants (27), the protective effect is likely related to the non-specific activation of innate immunity after influenza vaccination which would enhance the production of proinflammatory cytokine, triggering a non-specific protection against other viruses (28).

Previous reports highlighted the need to untangle the SARS-CoV-2 immunologic response differences in race/ethnicity based on the claim that Covid-19 infection is likely related to socioeconomic status, not race nor ethnicity (14, 15). A recent study reported that there are higher infection and mortality rates in counties with higher poverty rates and with more, Non-White, and more non diverse populations (17). In this project, we used global data from TriNetX; we did not assess the COVID-19 infection risks of patients in different socioeconomic backgrounds. However, the TriNetX cohort we explored reveals a higher incidence of COVID-19 infection among patients of African American/Black race. Because ethnicity was a separate variable and can be assessed separately from the “race” variable, we analyzed the data using the ethnicity variable as well. Surprisingly, we found no ethnicity differences (Hispanics vs. non-Hispanics) in the incidence of COVID-19 infection in children. Our study did not find significant gender differences in the incidence of COVID-19 infections. We found gender and ethnicity effects in COVID-19 infection incidence rates when stratified by weight categories.

Data on the relationship between COVID-19 infection and obesity in the pediatric population is scarce. Our study suggests that the overall incidences of COVID-19 infection increased statistically with increasing weight category—normal weight, overweight, and obese—but this finding is probably clinically insignificant because of our large sample size. However, obesity as a suggested risk factor for COVID-19 infection should be interpreted in the context of race and ethnicity, as we noticed a higher proportion of patients with COVID-19 of Native Hawaiian race, and of Hispanic ethnicity have and overweight or obesity diagnosis. Our findings are similar to a nationwide case-control study from South Korea, which included 3,788 COVID-19 cases and 15,152 matched controls; even after adjustment for sociodemographic, comorbidity, laboratory, and medication data, higher weight category levels were associated with a higher risk of contracting COVID-19 infections (18). However, this study did not report the race of the participant (18). It is anticipated that most of the patients in the South Korean cohort were Asian, and the number of participants is small compared to our study.

Our finding of a higher incidence of MIS-C among patients with higher BMI supports the previously published studies that obesity is associated with increased COVID-19 severity (5, 19–22). In addition, a study on 494 COVID-19-infected children suggested that the association between obesity and COVID-19 severity is age-dependent and that obesity increases the risk for COVID-19-related critical illness in adolescents but not in younger children (8). However, our findings do not support these findings. Interestingly, the same study reported that higher BMI was not associated with length of hospitalization, ICU admission, myocarditis, MIS-C, acute kidney injury, or mortality for any age (8). In our study, we only analyzed MIS-C as a sequela of COVID-19 infection and did not study the post-infectious single system involvement such as cardiac or renal systems.

To date, there are no pediatric reports about the association between prior Influenza vaccination, or both Influenza vaccination and COVID-19 vaccination, and the outcomes of COVID-19 infection. However, data from adult studies suggested that influenza vaccination might have a protective effect against COVID-19 infection and reports less severe acute illness presentation in previously influenza vaccinated adults (23–25). Our data uniquely demonstrate that influenza vaccination, COVID-19 vaccination, or both significantly improve the outcomes in children with COVID-19 infection. Using the multiplicative interaction scale, we found a positive interaction between the COVID-19 vaccine and influenza vaccine; they both combined have a more significant effect than each separately; the positive interaction is modest for hospitalization risk; however, the interaction was strong for MIS-C and mortality risk.

Our study has several limitations. First of all, our study has a potential bias having about 20%–22% of COVID-19 infected patients with unknown race or ethnicity, which possibly skewed the results towards higher incidence in one race or ethnicity and underestimated the incidence in other races or ethnicities, however giving the magnitude of the cohort, the potential of this skew is unlikely. Using the TriNetX system, we think that our analysis is limited due to building the cohort query based on supported code systems only. It is possible that many patients were missed if the correct code was not used during patient care. Another limitation of our study is that our methods do not let us identify the exact reason for hospitalization post COVID-19 infection. We used the ICD 10-codes to identify patients with normal weight, overweight, and obesity (*n* = 1,203,905). We did not use the actual weight or BMI of each patient. Therefore, our data did not include 90% of the total patients in the cohort (17,158,646). Nevertheless, the sample size of 1,203,905 patients with weight category, based on ICD encounter diagnoses, reported is large enough to draw an association between COVID-19 infection, race, and ethnicity, through the weight categories, and an association between weight categories and MIS-C risk. Another limitation of our work is that our cohorts were built based on a single dataset, which is approximately 64% from US, and approximately 23% from European, Middle Eastern and African countries all together. However, the dataset contained EHR data from over 100 million patients.

## Conclusion

Scientists have reached a considerable level of understanding of COVID-19 infection in children and are starting to understand MIS-C better. Further studies are needed to better understand the relationship between obesity and pediatric severe COVID-19 infection and post-infection sequela, including MIS-C. Our work displayed the synergistic effect of being vaccinated against both COVID-19 and influenza, which encourage researchers to develop a combined vaccine in the future. Future studies examining the immunologic responses of individuals to COVID-19 infection who were recently vaccinated by other viral infection vaccines are needed. Nevertheless, more extensive, and collaborative prospective studies are needed to understand the relation between COVID-19 illness and influenza vaccination and the potential relation between COVID-19 host defense and other vaccines, which could explain why the pediatric population has less severe COVID-19 infection and infection-related outcomes as compared to adults in general.

## Data Availability

The original contributions presented in the study are included in the article/**Supplementary Material**, further inquiries can be directed to the corresponding author.
