# Supplementary material for: Characteristics of pediatric COVID-19 infections and the impact of influenza and COVID-19 vaccinations during the first two years of the pandemic
Source: Front Pediatr. 2023 Oct 12;11:1046680. doi: 10.3389/fped.2023.1046680 (PMC10600516; doi:10.3389/fped.2023.1046680)
Supplement: Supplementary file 1 [file Datasheet1.pdf]

Supplementary Material

1 Supplementary Data

2 Appendix: TriNetX codes used to identify the variables and outcomes of interest

3 ICD-10-CM J12.81: Pneumonia due to SARS-associated coronavirus

4 ICD-10-CM J12.82: Pneumonia due to COVID-19

5 ICD-10-CM U07.1: COVID-19

6 ICD-10-CM U07.2: COVID-19, virus not identified (WHO)

7 ICD-10-CM B34.2: Coronavirus infection, unspecified

8 ICD-10-CM B97.29: Other coronavirus as the cause of diseases classified elsewhere

9 9089: SARS coronavirus 2 IgG IgM Ab [Presence] in Serum or Plasma

10 9088: SARS coronavirus 2 and related RNA [Presence]

11 94505-5: SARS coronavirus 2 IgG Ab [Units/volume] in Serum or Plasma by Immunoassay

12 94506-3: SARS coronavirus 2 IgM Ab [Units/volume] in Serum or Plasma by Immunoassay

13 94562-6 SARS coronavirus 2 IgA Ab [Presence] in Serum or Plasma by Immunoassay

14 94762-2: SARS coronavirus 2 Ab [Presence] in Serum or Plasma by Immunoassay

15 94769-7: SARS coronavirus 2 Ab [Units/volume] in Serum or Plasma by Immunoassay

16 94558-4: SARS coronavirus 2 Ag [Presence] in Respiratory specimen by Rapid Immunoassay

17 95209-3: SARS coronavirus + SARS coronavirus 2 Ag [Presence] in Respiratory specimen by Rapid immunoassay

18 96119-3: SARS-COV-2 (COVID-19) Ag [Presence] in Upper respiratory specimen by Immunoassay

19 96603-6: SARS-CoV-2 (COVID-19) S protein RBD neutralizing antibody [Presence] in Serum or Plasma by Immunoassay

20 94763-0: SARS-CoV-2 (COVID-19) [Presence] in Unspecified specimen by Organism specific culture

21 96742-2: SARS-COV-2 (COVID-19) IgG Ab [Mass/volume] in Serum or Plasma by Immunoassay

22 97097-0: SARS-COV-2 (COVID-19) Ag [Presence] in Upper respiratory specimen by Rapid immunoassay

23

24 95522-9: SARS-COV-2 (COVID-19) N gene [Log #volume] (viral load) in respiratory specimen by NAA with probe detection

25 88: Flu

26 90630: Influenza virus vaccine, quadrivalent (IIV4), split virus, preservative free, for intradermal use

27 90653: Influenza vaccine, inactivated (IIV), subunit, adjuvanted, for intramuscular use

28 90654: Influenza virus vaccine, trivalent (IIV3), split virus, preservative-free, for intradermal use98655; Influenza virus vaccine, trivalent (IIV3), split virus, preservative free, 0.25 mL dosage, for intramuscular use

29 90656: Influenza virus vaccine, trivalent (IIV3), split virus, preservative free, 0.5 mL dosage, for intramuscular use

30 90657: Influenza virus vaccine, trivalent (IIV3), split virus, 0.25 mL dosage, for intramuscular use

31 90658: Influenza virus vaccine, trivalent (IIV3), split virus, 0.5 mL dosage, for intramuscular use

32 90660: Influenza virus vaccine, trivalent, live (LAIV3), for intranasal use

33 90661; Influenza virus vaccine, trivalent (ccllV3), derived from cell cultures, subunit, preservative and antibiotic free, 0.5 mL dosage, for intramuscular use

34 90662: Influenza virus vaccine (IIV), split virus, preservative free, enhanced immunogenicity via increased antigen content, for intramuscular use

35 90664: Influenza virus vaccine, live (LAIV), pandemic formulation, for intranasal use

36 90666: Influenza virus vaccine (IIV), pandemic formulation, split virus, preservative free, for intramuscular use

37 90667: Influenza virus vaccine (IIV), pandemic formulation, split virus, adjuvanted, for intramuscular use

38 90668: Influenza virus vaccine (IIV), pandemic formulation, split virus, for intramuscular use

39 90672: Influenza virus vaccine, quadrivalent, live (LAIV4), for intranasal use

40 90673: Influenza virus vaccine, trivalent (RIV3), derived from recombinant DNA, hemagglutinin (HA) protein only, preservative and antibiotic free, for intramuscular use

41 90674: Influenza virus vaccine, quadrivalent (cclIV4), derived from cell cultures, subunit, preservative and antibiotic free, 0.5 mL dosage, for intramuscular use

42 98682: Influenza virus vaccine, quadrivalent (RIV4), derived from recombinant DNA, hemagglutinin (HA) protein only, preservative and antibiotic free, for intramuscular use

43 90685: Influenza virus vaccine, quadrivalent (IIV4), split virus, preservative free, 0.25 mL dosage, for intramuscular use

44 90686: Influenza virus vaccine, quadrivalent (IIV4), split virus, preservative free, 0.5 mL dosage, for intramuscular use

45 90687: Influenza virus vaccine, quadrivalent (IIV4), split virus, 0.25 mL dosage, for intramuscular use

46 90688: Influenza virus vaccine, quadrivalent (IIV4), split virus, 0.5 mL dosage, for intramuscular use

47 90689: Influenza virus vaccine, quadrivalent (IIV4), inactivated, adjuvanted, preservative free, 0.25 mL dosage, for intramuscular use

48 90694: Influenza virus vaccine, quadrivalent (allV4), inactivated, adjuvanted, preservative free, 0.5 mL dosage, for intramuscular use

49 90756: Influenza virus vaccine, quadrivalent (ccIIV4), derived from cell cultures, subunit, antibiotic free, 0.5 mL dosage, for intramuscular use

50 Q2034; Influenza virus vaccine, split virus, for intramuscular use (agriflu)

51 Q2035: Influenza virus vaccine, split virus, when administered to individuals 3 years of age and older, for intramuscular use (afluria)

52 Q2036; Influenza virus vaccine, split virus, when administered to individuals 3 years of age and older, for intramuscular use (flulaval)

53 Q2037: Influenza virus vaccine, split virus, when administered to individuals 3 years of age and older, for intramuscular use (fluvirin)

54 Q2038: Influenza virus vaccine, split virus, when administered to individuals 3 years of age and older, for intramuscular use (fluzone)

55 Q2039: Influenza virus vaccine, not otherwise specified

56 G0008 Administration of influenza virus vaccine

57 3E02340: Introduction of Influenza Vaccine into Muscle, Percutaneous Approach

58 3E01340: Introduction of Influenza Vaccine into Subcutaneous Tissue, Percutaneous Approach

59 86198006: Administration of influenza vaccine

60 Z68.52: Body mass index [BMI] pediatric, 5th percentile to less than 85th percentile for age

61 Z68.53: Body mass index [BMI] pediatric, 85th percentile to less than 95th percentile for age

62 Z68.54: Body mass index [BMI] pediatric, greater than or equal to 95th percentile for age

63 M35.81: Multisystem inflammatory syndrome (MIS-C)

64 Visit: inpatient encounter: Admission

65 Visit: inpatient non-acute: Admission

66 Visit: Short stay: Admission

67 CPT#1013659: Hospital inpatient services

68 CPT#1013699: Initial inpatient consultation services

69 CPT#1013729: Critical care services

70 Deceased: Mortality

71 213: SARS-CoV-2 (COVID-19) Vaccine

72 91300: severe acute respiratory syndrome coronavirus 2 (SARS-CoV-2) (Coronavirus disease (COVID-191) vaccine, mRNA-LNP spike protein, preservative free, 30 mcg/0.3mL dosage, diluent reconstituted, for intramuscular use

73 91301: severe acute respiratory syndrome coronavirus 2 (SARS-CoV-2) (Coronavirus disease (COVID-19]) vaccine, mRNA-LNP, spike protein, preservative free, 100 mcg/0.5ml dosage, for intramuscular use

74 91302: severe acute respiratory syndrome coronavirus 2 (SARS-CoV-2) (coronavirus disease (COVID-19]) vaccine, DNA, spike protein, chimpanzee adenovirus Oxford 1 (ChAdOx1) vector, preservative free, 5x10^10 viral particles/0.5ml dosage, for intramuscular use

75 91303: severe acute respiratory syndrome coronavirus 2 (SARS-CoV-2) (coronavirus disease [COVID-19]) vaccine, DNA, spike protein, adenovirus type 26 (Ad26) vector, preservative free, 5x10^10 viral particles/0.5ml dosage, for intramuscular use

76 Xw013S6: Introduction of COVID-19 Vaccine Dose 1 into Subcutaneous Tissue, Percutaneous Approach, New Technology Group 6

77 Xw013T6: Introduction of COVID-19 Vaccine Dose 2 into Subcutaneous Tissue, Percutaneous Approach, New Technology Group 6

78 Xw013U6: Introduction of COVID-19 Vaccine into Subcutaneous Tissue, Percutaneous Approach, New Technology Group 6

79 XW023S6: Introduction of COVID-19 Vaccine Dose 1 into Muscle, Percutaneous Approach, New Technology Group 6

80 Xw023T6: Introduction of COVID-19 Vaccine Dose 2 into Muscle, Percutaneous Approach, New Technology Group 6

81 xw023U6: Introduction of COVID-19 Vaccine into Muscle, Percutaneous Approach, New Technology Group 6

82 840534001: severe acute respiratory syndrome coronavirus 2 vaccination

83 91305: severe acute respiratory syndrome coronavirus 2 (SARS-COV-2) (coronavirus disease (COVID-19]) vaccine, mRNA-LNP, spike protein, preservative free, 30 mcg/0.3 mL dosage, tris-sucrose formulation, for intramuscular use

84 91307: severe acute respiratory syndrome coronavirus 2 (SARS-COV-2) (coronavirus disease [COVID-19]) vaccine, mRNA-LNP, spike protein, preservative free, 10 mcg/0.2 mL dosage, diluent reconstituted, tris sucrose formulation, for intramuscular use

85 91306: severe acute respiratory syndrome coronavirus 2 (SARS-COV-2) (coronavirus disease [COVID-19]) vaccine, mRNA-LNP, spike protein, preservative free, 50 mcg/0.25 mL dosage, for intramuscular use

86 91308: severe acute respiratory syndrome coronavirus 2 (SARS-COV-2) (coronavirus disease [COVID-19]) vaccine, mRNA-LNP, spike protein, preservative free, 3 mcg/0.2 mL dosage, diluent reconstituted, tris sucrose formulation, for intramuscular use

87 1119350007: Administration of SARS-CoV-2 mRNA vaccine

88 1144997007: Administration of first dose of SARS-COV-2 mRNA vaccine

89 1144998002: Administration of second dose of SARS-COV-2 mRNA vaccine

90 1156257007: Administration of SARS-CoV-2 vaccine

91 1157108008: Administration of second dose SARS-CoV-2 non-replicating viral

92 1157187003: Administration of SARS-CoV-2 non-replicating viral vector vaccine

93 1157196000: Administration of inactivated whole SARS-CoV-2 antigen vaccine OR

94 1157197009: Administration of second dose of inactivated whole SARS-COV-2 antigen vaccine

95 1162645008: Administration of SARS-CoV-2 recombinant spike protein antigen vaccine

96 1162646009: Administration of second dose of SARS-CoV-2 recombinant spike protein antigen vaccine

Supplementary Figures and Tables

<u>Table: BMI Distribution across pediatrics patients who did not have COVID-19 diagnoses</u>

BMI reported and its distribution in patients without COVID-19 diagnosis (n=878,565)		BMI between 5th and < 85th percentile for age n (%)	BMI between 85th and < 95th percentile for age n (%)	BMI ≥ 95th percentile for age n (%)
N of cohort per BMI (total n = 878,565)		429,419 (48.88%)	163,123 (18.57%)	286,028 (32.56%)
Gender	Female (total n = 428,039)	210,413 (49.16%)	83,193 (19.43%)	134,433 (31.41%)
	Male (total n = 450,526)	219,001 (48.61%)	79,930 (17.74%)	151,595 (33.65%)
	Unknown (total n = 0)	0 (0%)	0 (0%)	0 (0%)
Race	American Indian or Alaskan native (total n = 4,491)	0 (0%)	1,631 (36.32%)	2,860 (63.68%)
	Asian (total n = 36,577)	21,471 (58.7%)	6,525 (17.84%)	8,581 (23.46%)
	African American/Black (total n = 178,937)	77,294 (43.2%)	35,887 (20.06%)	65,756 (36.75%)
	Native Hawaiian or other pacific islander (total n = 0)	0 (0%)	0 (0%)	0 (0%)
	Unknown (total n = 222,501)	107,353 (48.25%)	40,781 (18.33%)	74,367 (21.29%)
	White (total n = 436,030)	223,298 (51.21%)	78,299 (17.96%)	134,433 (30.83%)
Ethnicity	Hispanic or Latino (total n = 151,170)	51,530 (34.09%)	30,993 (20.5%)	68,647 (45.41%)
	Not Hispanic/ Not Latino (total n = 498,972)	266,240 (53.36%)	89,718 (17.98%)	143,014 (28.66%)
	Unknown (228,428)	111,649 (48.88%)	42,412 (18.57%)	74,367 (32.55%)
